# Supplementary material for: Iron mineral dissolution releases iron and associated organic carbon during permafrost thaw
Source: Nat Commun. 2020 Dec 10;11:6329. doi: 10.1038/s41467-020-20102-6 (PMC7729879; doi:10.1038/s41467-020-20102-6)
Supplement: Supplementary file 1 — Supplementary Information [file 41467_2020_20102_MOESM1_ESM.pdf]

Supplementary Information for

Iron mineral dissolution releases iron and associated organic carbon during permafrost thaw

By Patzner et al.

Supplementary Note 1

Replication along a thaw gradient

To evaluate the consistency of iron and carbon trends along the thaw gradient, 5 cores per thaw stage were analyzed in total. These include:

(1) Palsa A, Bog C and Fen E in 2018 (discussed in the main text) (1 core per thaw stage)

Cores were taken with a Humax corer and sterile plastic liners in June 2018. This set was immediately split and processed after sampling (3-4 days) (Supplementary Figure 1, a, yellow cores).

(2) Palsa a, Bog c and Fen e in 2019 (1 core per thaw stage)

These cores were taken with a Humax corer in July 2019 (Supplementary Figure 1, a, red). Also, this set of cores was immediately split and processed after sampling and thus is directly comparable to Palsa A, Bog C and Fen E.

(3) Palsa B, Bog D and Fen F in 2018 (1 core per thaw stage)

This set was taken at the same time and same conditions as cores Palsa A, Bog C and Fen E, but stored at 4°C for 7 months and then processed (Supplementary Figure 1, a, white cores).

(4) Triplicate cores in each thaw stage in 2017 (3 cores per thaw stage)

Triplicate cores in each thaw stage were taken with a Pürckhauer corer in September 2017 and immediately processed after sampling (Supplementary Figure 1, a, orange, green and blue cores). The whole depth profile was successfully captured for all palsa triplicate cores. The organic horizon was lost for the bog and the fen triplicate cores during coring, thus are not reported in Supplementary Figure 4 and Supplementary Figure 5. In bog and fen, the transition zones and mineral horizons were successfully sampled, confirming the loss of reactive iron along the thaw gradient at several spots in the permafrost peatland mire.

All cores showed the same trend for total extractable iron, for the poorly crystalline iron and the total carbon along the thaw gradient. However, we did not directly combine these observations in the main text as there was some variation in the sampling and storage methods.

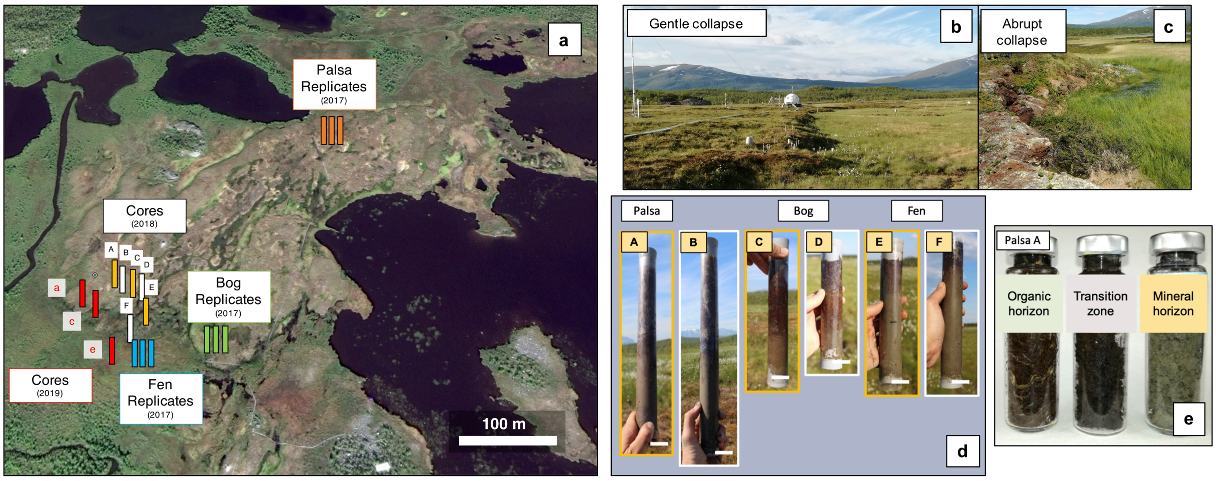

Supplementary Figure 1. Coring at Stordalen mire, Abisko (Sweden). a, Position of cores taken along a thaw gradient at Stordalen mire (Abisko, Sweden). Yellow: Cores were immediately split and processed after sampling (3-4 days). White: Cores were stored at 4°C for 7 months and then processed. Red: Cores were immediately split and processed after sampling in the same manner as the cores in yellow (palsa a (68°21'18.03"N, 19° 2'35.20"E), bog c (68°21'18.02"N, 19° 2'36.53"E) and fen e (68°21'17.24"N, 19° 2'37.44"E)). The data was compared to triplicate cores in each thaw stage (palsa in orange, bog in green and fen in blue). b, Gentle collapse of palsa sites at Stordalen mire (Abisko, Sweden). The sampled thaw gradient represents a gentle collapse of palsa to bog. c, Abrupt collapse of palsa sites at Stordalen mire (Abisko, Sweden). Permafrost thaw does not necessarily progress through all three thaw stages (palsa, bog and fen). d, Cores taken along a thaw gradient. Palsa: Core A (68°21'18.70"N, 19° 2'38.00"E) and core B (68°21'18.50"N, 19° 2'38.80"E) showed the three main layers in the palsa area: (1) organic horizon, (2) transition zone and (3) mineral horizon. The organic layer was dry and oxic. Bog: Core C (68°21'18.60"N, 19° 2'39.20"E) and core D (68°21'18.30"N, 19° 2'40.00"E) clearly showed the division into the three layers: (1) organic horizon, (2) transition zone and (3) mineral horizon. Both cores were completely water saturated. Fen: Core E (68°21'16.80"N, 19° 2'40.30"E) and core F (68°21'17.80"N, 19° 2'41.30"E) also showed the three layers and were water saturated. The cores represent the active layer in July 2018. The scale bar represents 3 cm. e, Example of the subdivision into (1) organic horizon, (2) transition zone and (3) mineral horizon (Palsa A).

(1) Replicate cores Palsa a, Bog c and Fen e (in 2019)

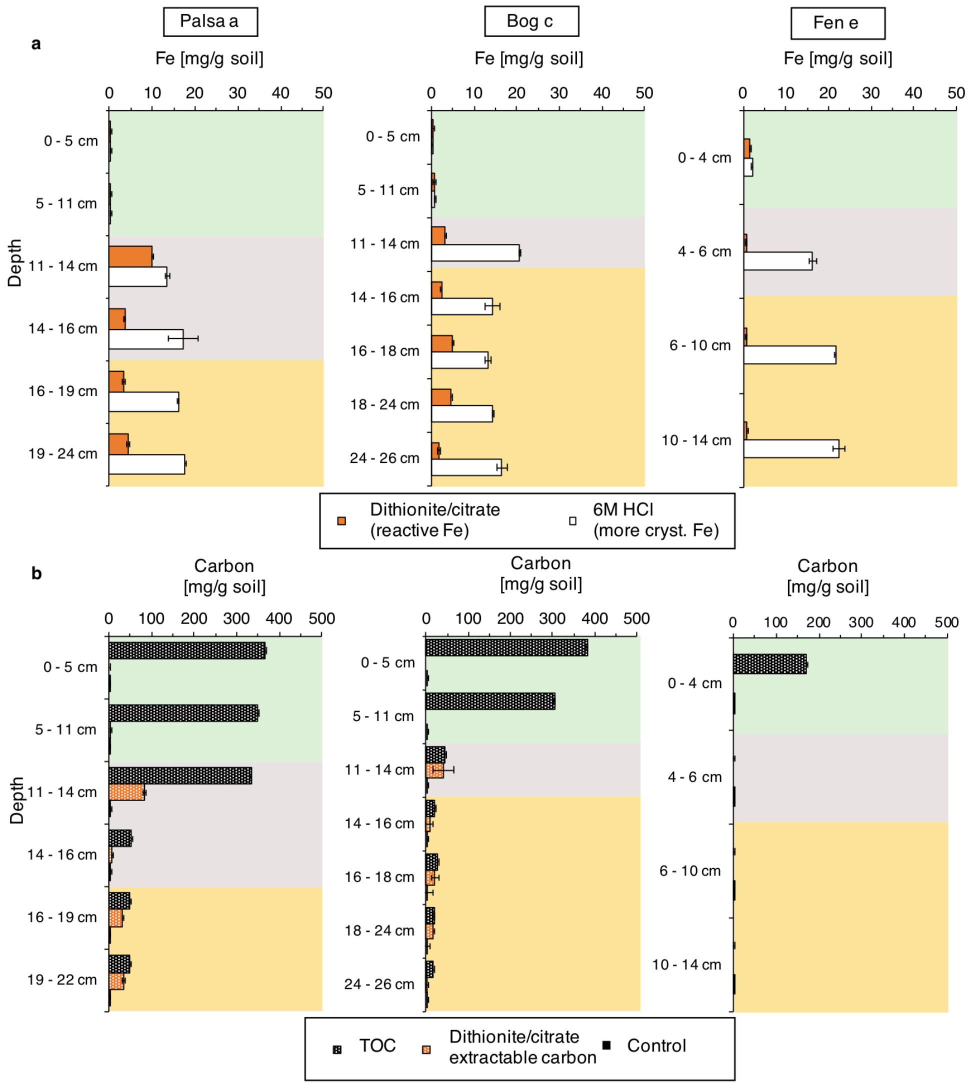

Supplementary Figure 2. Extractions of replicate cores. a, Iron and b, Carbon concentration of cores Palsa a, Bog c and Fen e. Cores were taken in July 2019, split directly after sampling and immediately processed. The green box marks the organic horizon, grey box the transition zone and yellow box the mineral horizon. Extractable iron was determined via the ferrozine assay. Total organic carbon (TOC) was determined via combustion, whereas the carbon in the dithionite citrate (control corrected) and the control extract (sodium chloride bicarbonate) was determined with the carbon analyzer. Errors of the TOC and 6M hydrochloric acid (HCl) extractable iron indicate the range of duplicate analyses of each layer in each thaw stage. Errors of the dithionite/citrate extractable a, iron and b, carbon (control corrected) represent a combined standard deviation of sodium chloride bicarbonate extractable a, iron and b, carbon, b, citrate blank and dithionite/citrate extractable a, iron and b, carbon (not control corrected).

(2) Replicate cores Palsa B, Bog D, Fen F along the thaw gradient (in 2018)

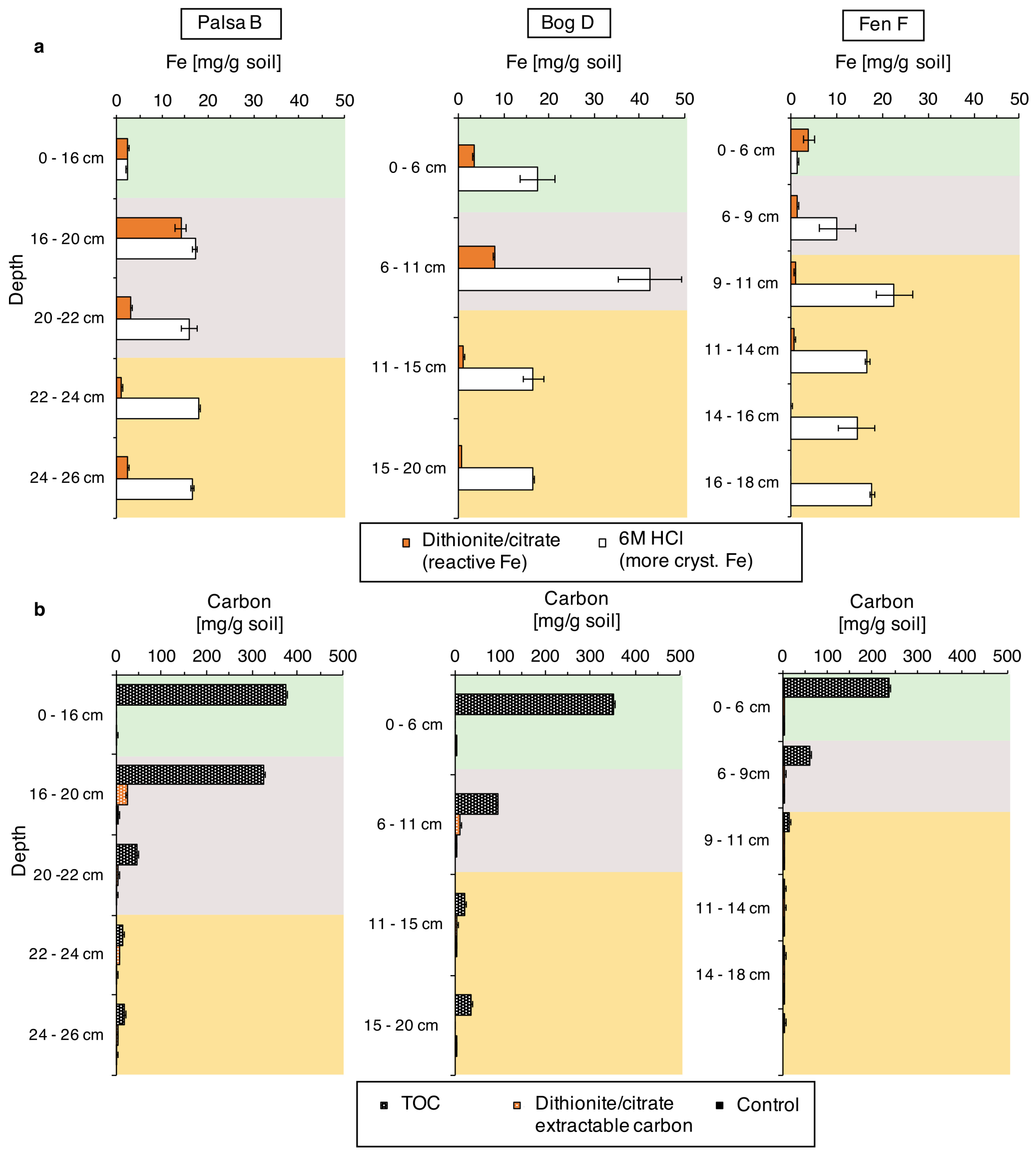

Supplementary Figure 3. Extractions of replicate cores. a, Iron and b, Carbon concentration of cores Palsa B, Bog D and Fen F. Cores were split after 7 months of incubation at 4°C. The green box marks the organic horizon, grey box the transition zone and yellow box the mineral horizon. Iron in the extracts was determined via the ferrozine assay. Total organic carbon (TOC) was determined via combustion, whereas the carbon in the dithionite citrate (control corrected) and the control extract (sodium chloride bicarbonate) was determined with the carbon analyzer. Errors of the TOC and 6M hydrochloric acid (HCl) extractable iron indicate the range of duplicate analyses of each layer in each thaw stage. Errors of the dithionite/citrate extractable a, iron and b, carbon (control corrected) represent a combined standard deviation of sodium chloride bicarbonate extractable a, iron and b, carbon, b, citrate blank and dithionite/citrate extractable a, iron and b, carbon (not control corrected).

(3) Triplicate cores in each thaw stage (iron analysis) (in 2017)

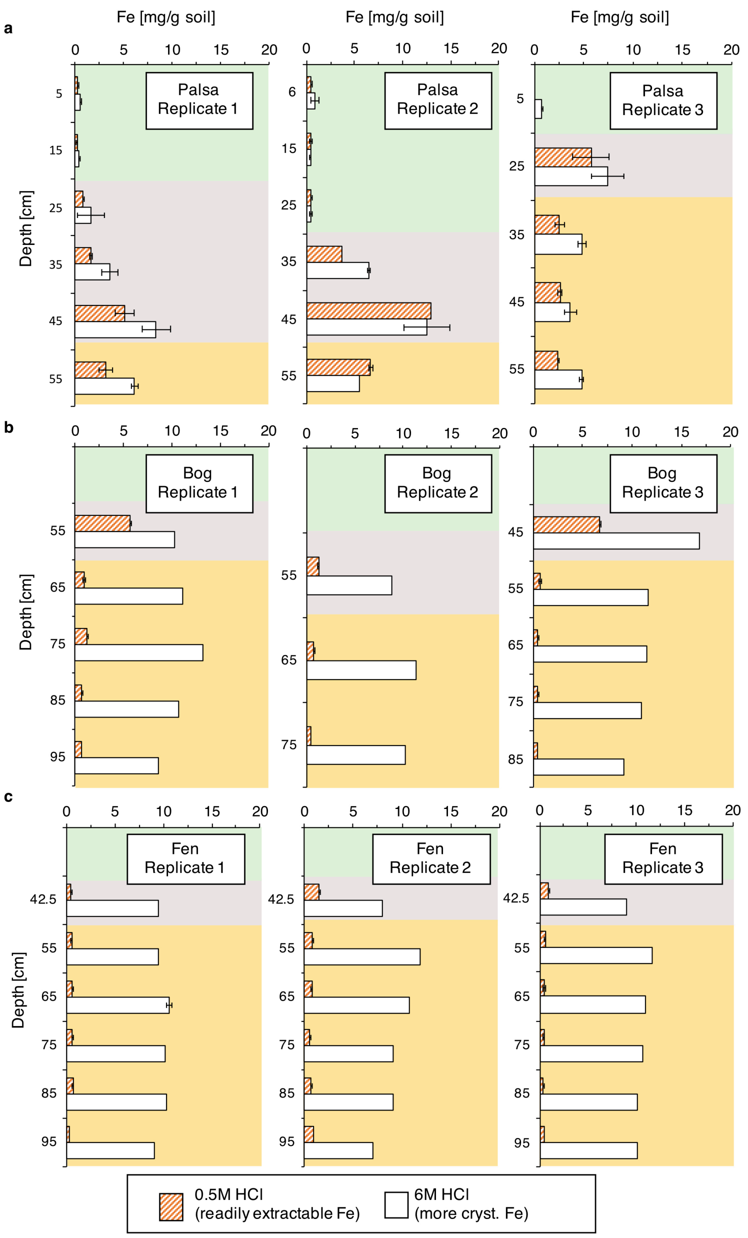

Supplementary Figure 4. Iron extractions of replicate cores taken with a Pürckhauer corer: a, Palsa (68°21'26.56"N, 19° 3'0.19"E), b, Bog (68°21'16.02"N, 19° 2'49.21"E), c, Fen (68°21'17.16"N, 19° 2'36.29"E). Each core was divided into layers in the field and immediately processed. The organic horizon for the triplicate cores of bog and fen were lost during sampling and thus, are not reported in this figure. All replicates represent the active layer in September 2017. The bog and the fen soils were waterlogged. The green box marks the organic horizon, grey box the transition zone and yellow box the mineral horizon. Errors indicate the range of duplicate analyses of each layer in each thaw stage.

(4) Triplicate cores in each thaw stage (carbon analysis)

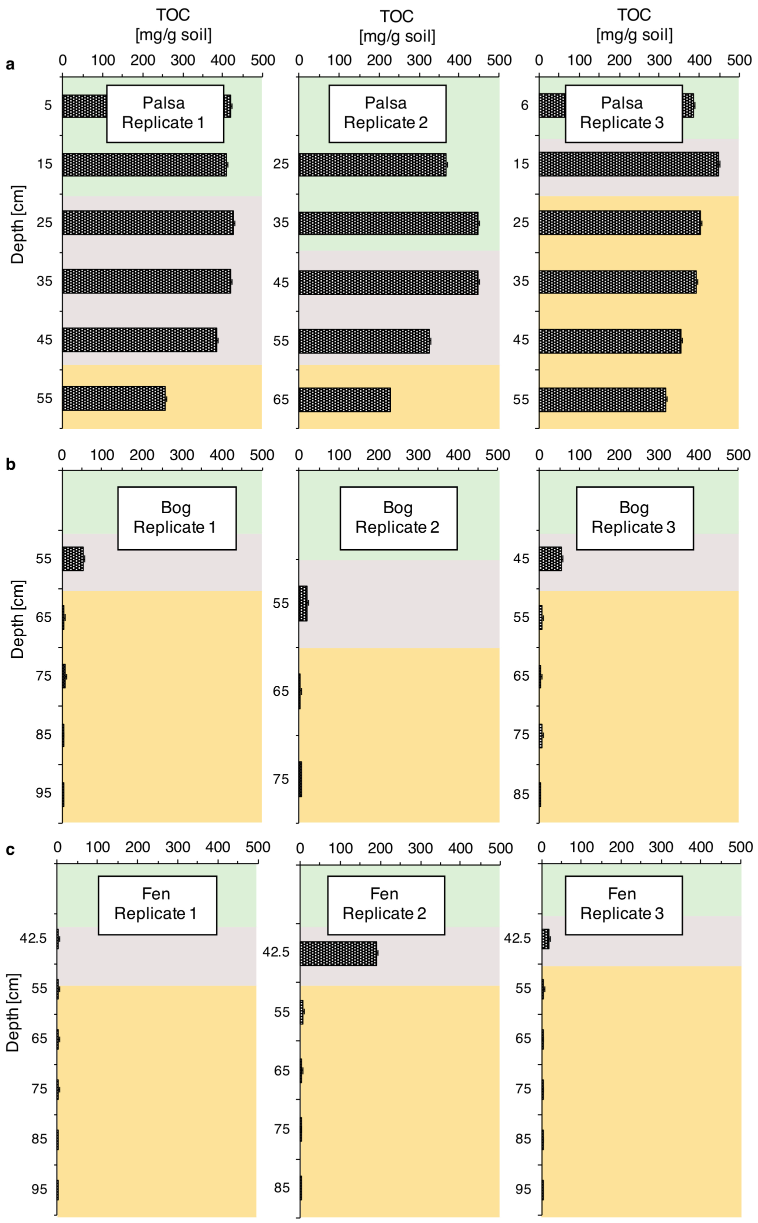

Supplementary Figure 5. TOC of replicate cores taken with a Pürckhauer corer: a, Palsa (68°21'26.56"N, 19° 3'0.19"E), b, Bog (68°21'16.02"N, 19° 2'49.21"E) and c, Fen (68°21'17.16"N, 19° 2'36.29"E). Each core was divided into layers in the field and immediately processed. The organic horizon for the bog and fen triplicate cores were lost during sampling and thus are not reported in this figure. All replicates represent the active layer in September 2017. The bog and fen soils were waterlogged. The green box marks the organic horizon, grey box the transition zone and yellow box the mineral horizon. Total organic carbon (TOC) was determined via combustion. Errors indicate the range of duplicate analyses of each layer in each thaw stage.

Supplementary Figure

Fe(III) reduction by isolated Fe(III)-reducing bacteria from the fen, consuming lactate and forming acetate

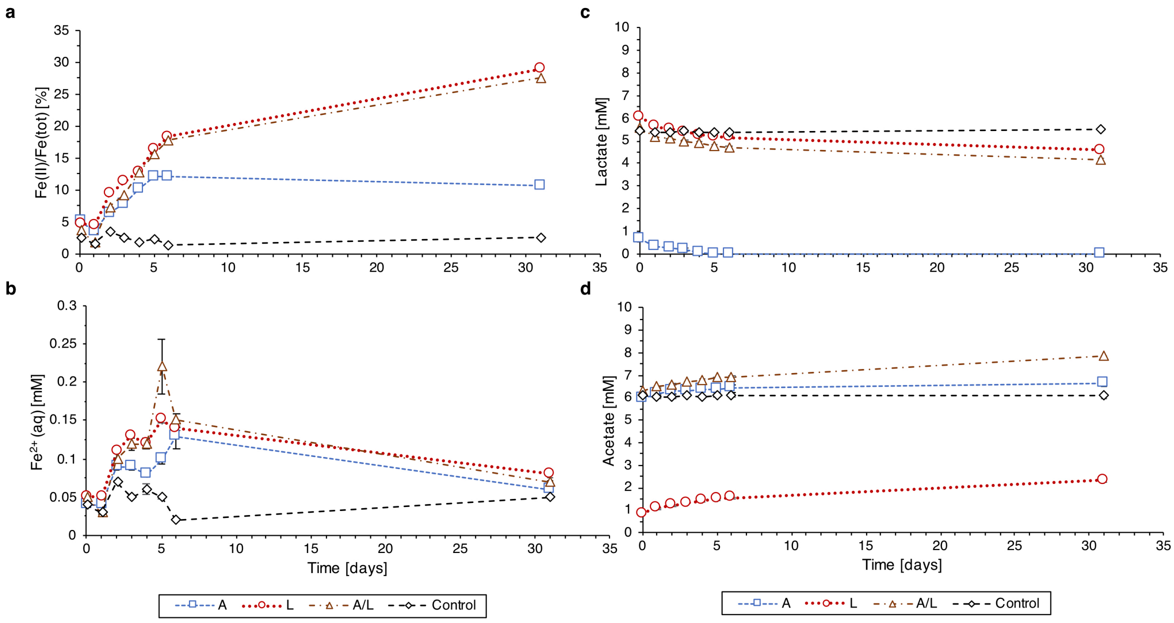

Supplementary Figure 6. Iron (Fe) (III) reduction by the Fe(III) reducer isolate from the fen soil core in four experimental setups: only acetate (A), only lactate (L), both acetate and lactate (A/L) and control (no amendments added). a, Fe(II) to Fe(tot) ratio in % in the solid phase over time. 100% would mean that all initial present ferrihydrite was reduced to Fe(II). b, Fe(II) released into the liquid phase over time. c, lactate consumption over time. d, acetate formation over time. Note: The culture which was used for inoculum was previously cultivated in medium supplemented with 5 mM acetate and 5 mM lactate. Therefore, approximately 1 mM of both organic acids were transferred together with the bacteria in all setups, leading to minor Fe(III) reduction also in the acetate only setup using the transferred lactate and forming acetate (0.6 mM) due to residual lactate consumption. After, 31 days, 25% of the initial Fe(III) was reduced to Fe(II) in the solid phase in the lactate only (L) and lactate and acetate (A/L) setup. The lactate concentrations decreased, and the acetate concentration increased by 1.5 mM over 31 days in the lactate amended (L) and (A/L) setup. Some Fe^{2+} was first released into the aqueous phase but later re-adsorbed onto iron minerals, resulting in a decrease in the aqueous Fe^{2+}. Error bars represent standard deviations from 3 batch bottles from 0 to 31 days. Each microcosm was measured in triplicates.

Supplementary Table

Absolute and % values of organic carbon and reactive iron content reported in the main text

Supplementary Table 1. Absolute and % values of iron and carbon in locations Palsa A, Bog C and Fen E, i.e. the cores reported in the main text. In most of the layers, the maximum mass ratio of organic carbon (OC) to iron (Fe) (reactive Fe-associated organic carbon:reactive Fe, OC:Fe (wt:wt)) exceeds 0.22, the maximal sorption capacity of reactive iron oxides for natural organic matter[1,2]. Co-precipitation and/or chelation of organic compounds can generate structures with OC:Fe ratios (wt:wt) above 0.22, as shown in other studies[1,2.] Errors of control iron, control carbon, total organic carbon and total extractable iron indicate the range of duplicate analyses of each layer in each thaw stage. Total extractable iron represents the 6M HCl extractable iron (more crystalline Fe phases). Errors of the dithionite/citrate extractable a, iron (reactive Fe, control corrected) and b, carbon (carbon bound to reactive iron, control corrected) represent a combined standard deviation of sodium chloride bicarbonate extractable a, iron and b, carbon, b, citrate blank and dithionite/citrate extractable a, iron and b, carbon (not control corrected).

	Reactive iron (control corrected)	Reactive iron of total extractable iron	Control iron	C bound to reactive iron (control corrected)	C bound to reactive iron of the total organic carbon	Control carbon	OC:Fe (wt:wt)	Total organic carbon	Total extractable Fe
	mg/g	%	mg/g	mg/g	%	mg/g		mg/g	mg/g
Palsa A									
Organic horizon	0.40 ± 0.11	100.00	0.00 ± 0.00	0.94 ± 0.58	0.22	1.37 ± 0.01	2.35	423 ± 0.00	0.20 ± 0.02
	0.29 ± 0.09	100.00	0.00 ± 0.00	2.16 ± 0.95	0.51	1.65 ± 0.08	7.45	422.91 ± 0.13	0.17 ± 0.00
Transition zone	2.55 ± 0.57	72.86	0.29 ± 0.08	30.99 ± 0.71	9.93	3.13 ± 0.02	12.15	312.11 ± 0.33	3.51 ± 0.08
	8.44 ± 0.21	93.86	0.75 ± 0.11	52.50 ± 0.13	14.80	10.36 ± 0.50	6.22	354.72 ± 0.04	8.99 ± 0.28
Mineral horizon	3.17 ± 0.19	36.58	0.25 ± 0.03	27.39 ± 1.61	20.13	2.88 ± 0.08	8.64	136.11 ± 0.21	8.65 ± 0.28
	1.35 ± 0.21	10.00	0.07 ± 0.06	13.58 ± 0.42	18.67	1.39 ± 0.10	10.06	72.71 ± 0.29	13.48 ± 0.22
Bog C									
Organic horizon	1.48 ± 0.18	40.60	0.73 ± 0.07	16.16 ± 3.91	4.85	3.16 ± 1.67	10.92	333.31 ± 0.05	3.63 ± 0.05
Transition zone	2.08 ± 0.05	11.14	0.41 ± 0.04	22.67 ± 8.60	39.42	1.18 ± 0.21	10.90	57.51 ± 0.38	18.65 ± 0.70
Mineral horizon	0.88 ± 0.06	7.52	0.28 ± 0.04	0.00 ± 0.00	0.00	1.04 ± 0.01	0.00	8.28 ± 0.25	11.69 ± 0.81
Fen E									
Organic horizon	2.03 ± 0.14	43.39	0.75 ± 0.00	0.00 ± 0.00	0.00	1.53 ± 0.00	0.00	234.70 ± 0.83	4.68 ± 0.01
Transition zone	2.64 ± 0.03	18.29	0.37 ± 0.00	0.00 ± 0.00	0.00	1.38 ± 0.18	0.00	16.24 ± 0.18	14.46 ± 0.22
Mineral horizon	1.75 ± 0.04	10.71	0.15 ± 0.00	0.00 ± 0.00	0.00	2.57 ± 0.76	0.00	3.52 ± 0.05	16.34 ± 0.44
	1.70 ± 0.04	8.95	0.19 ± 0.01	0.00 ± 0.00	0.00	1.13 ± 0.17	0.00	4.99 ± 0.10	19.01 ± 0.25

Supplementary Method 1

Fe-associated organic carbon: extraction method and controls

The determination of Fe-associated organic carbon has several well-known difficulties which can only be addressed by combining different approaches.

Considerations for the sodium dithionite-citrate extraction:

(1) *pH*

To prevent hydrolysis of organic matter as well as its protonation and re-adsorption onto sediment particles, which occur under acidic conditions, the sodium dithionite citrate extraction was performed at circumneutral pH (sodium bicarbonate buffered). Therefore, the additional hydroxylamine-HCl extraction (performed below pH 2) can only be a comparison for the sodium dithionite citrate extractable Fe, but not for the sodium dithionite citrate extractable carbon. The control extraction was performed under the same ionic strength (addition of NaCl) and pH (sodium bicarbonate buffered).

(2) *Temperature and incubation time*

Dithionite citrate bicarbonate extractions have been widely applied in various studies and were previously performed under two different temperatures and incubation times. One is conducted at room temperature at pH 7-8 for 16 hours on a shaker[1,3-6] and the other conducted at 80°C for 15 minutes[7,8]. Due to the high organic carbon content of the soil samples, the standard approach at room temperature at neutral pH for 16 hours was chosen to avoid alteration of carbon during heating of sample to 80°C, which could further influence the amount of extracted iron. The approach conducted at 80°C is suspected to contribute more to nonselective dissolution[9] in organic-rich samples.

(3) *Leaching of carbon which is not associated with iron*

The carbon measured in the sodium dithionite citrate extraction was corrected by subtraction of the measured DOC values in a citrate blank and in a control extraction, performed under the same ionic strength and pH. This control extraction determines how much carbon would be leached from the soil without any reduction (see Table S1 and Supplementary Figure 7). The effect of a reducing agent, which potentially reductively transforms certain organic functional groups, is not considered in this control (see point *(4)*).

(4) Dithionite as strong reducing agent

Dithionite is a strong reducing agent which can reductively transform certain organic functional groups and could lead to organic carbon release which is not associated with reactive Fe. Nevertheless, we consider this to be negligible for our extractions as the concentration of sodium dithionite citrate extractable carbon of a horizon containing primarily organic material and no mineral phase (Palsa A, organic horizon) is very low (0.3±0.1 to 0.4±0.1 mg extractable carbon per g soil; see also Supplementary Table 1). Additionally, a sodium pyrophosphate extraction was performed to determine the colloidal/OM-Fe. The sodium pyrophosphate extraction carbon yielded similar concentrations and showed similar trends to the dithionite-citrate extractable carbon across the thaw gradient (Supplementary Figure 7). Variation between the absolute values can occur due to heterogeneity in the samples and the alkaline conditions of the sodium pyrophosphate extraction (pH 10).

(5) Citrate as strong metal complexing agent

Citrate is a strong metal complexing agent that can influence the amount of extractable iron during dithionite extraction. A test-run was performed with the same experimental conditions (same ionic strength and pH), but no citrate addition. Without citrate, we obtained 64±3% less iron and 57±28% less carbon after sodium dithionite reductive dissolution. We therefore concluded, that the metal ion complexing agent citrate is necessary to avoid under-estimation of iron and organic carbon as a result of complexing or mineral precipitation during extraction.

Since extractions have well-known limitations as mentioned above, additional approaches were used and combined to characterize Fe-C associations in the solid phase along the thaw gradient.

Additional approaches used were:

(A) Extended X-ray absorption fine structure (EXAFS) with reference for reactive Fe (ferrihydrite) and references for organic carbon associated with Fe (Fe(II)-citrate) and Fe(III)-citrate)

(B) Nanoscale analysis using correlative SEM and nanoSIMS to show close spatial distribution of iron and carbon

The data set is consistent with an increase in aqueous Fe^{2+} and DOC and an increasing abundance of Fe(III)-reducing bacteria along the thaw gradient.

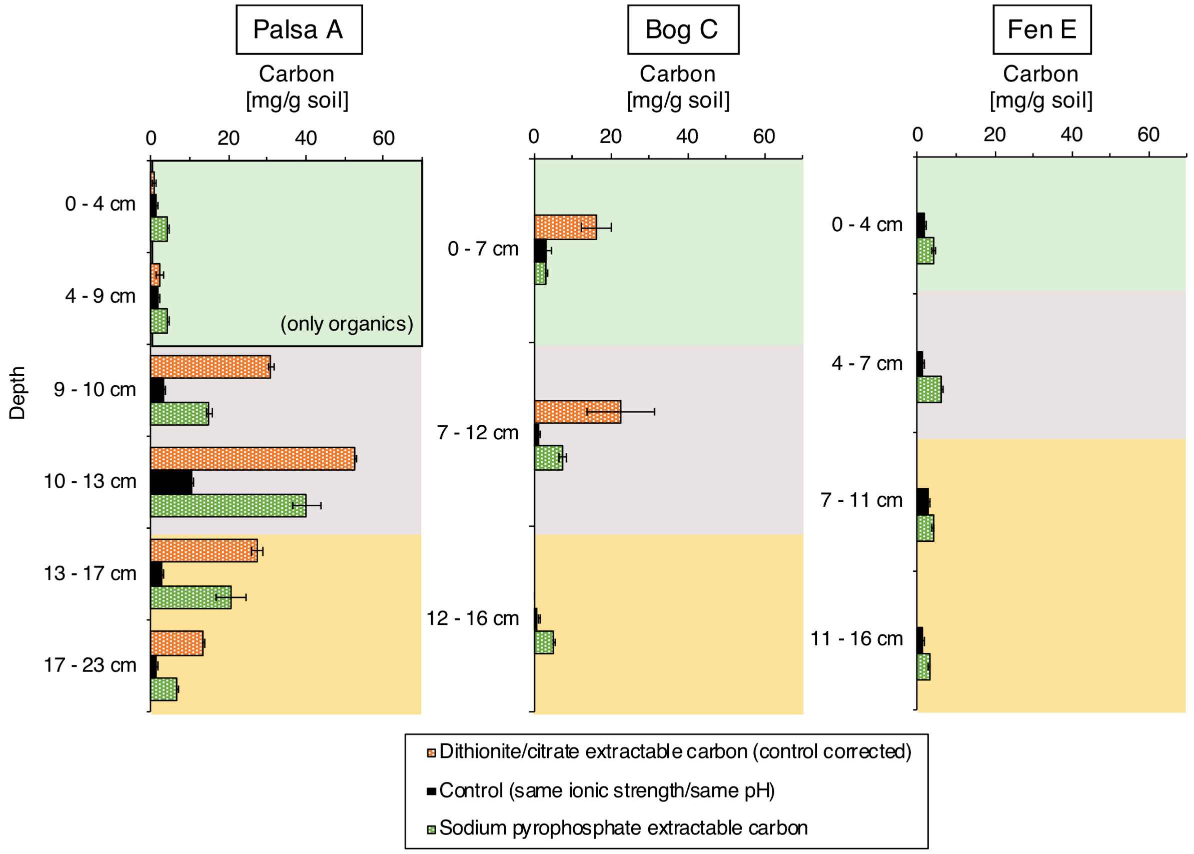

Supplementary Figure 7. Extractable carbon measured using different approaches to address issues with the sodium dithionite citrate extraction. The sodium dithionite citrate extractable carbon is control corrected (subtraction of citrate blank and extractable carbon of the control extraction with same ionic strength and pH), shown in orange (see also Supplementary Table 1). The control extraction (same ionic strength and pH) is shown in black (see also Supplementary Table 1). The sodium pyrophosphate (pH 10) extractable carbon shows similar amounts and trends with depth and along the thaw gradient, as the control corrected dithionite citrate extractable carbon. Dithionite citrate extractable carbon only from organics (Box, Palsa A, organic horizon) is low, therefore hydrolytic cleavage of organic matter can be ruled out. Errors indicate the range of duplicate analyses of each layer in each thaw stage.

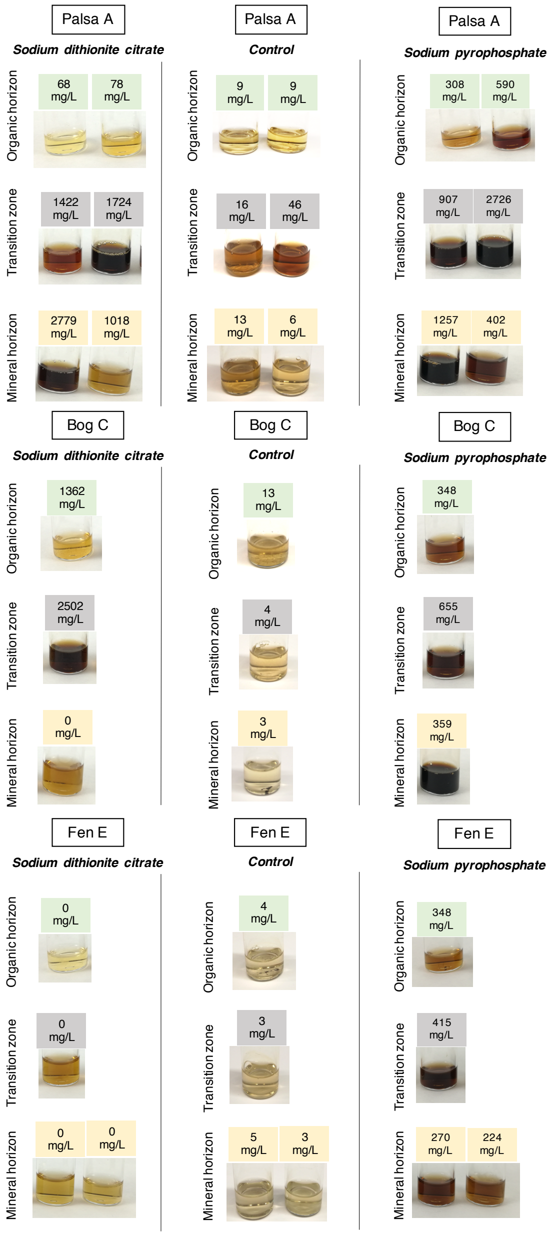

Supplementary Figure 8. Extracts of sodium dithionite, control and sodium pyrophosphate. Values of sodium dithionite citrate indicate the dissolved organic carbon concentrations in the extracts after subtraction of the background of the citrate concentration. The background concentration of the citrate for the analysis Palsa A, Bog C and Fen F was 20.5±0.14 g/L. Pictures of the extracts are shown to highlight the loss of the reactive iron phase and carbon associated with it along the thaw gradient, here visible by brown color loss in the sodium dithionite citrate extracts from palsa to fen. The control is shown to demonstrate that there was no carbon leached under same ionic strength and pH. The sodium pyrophosphate extracts support the trends shown by the sodium dithionite citrate extraction. Green marks the organic horizon, grey the transition zone and yellow the mineral horizon.

Supplementary Note 2

Calculated stocks (mg/cm^2) versus content (mg/g) of reactive Fe and reactive Fe-associated organic carbon

During coring compaction occurred. Based on the bulk densities, compaction was assumed to have occurred in the palsa organic horizon (0.03±0.01 g/cm^3) and palsa transition zone (0.08±0.02 g/cm^3), but not for the dense palsa mineral horizon (0.84±0.26 g/cm3). For the bog organic horizon (0.08±0.01 g/cm^3) and for fen organic horizon (0.21±0.02 g/cm^3), compaction was assumed but we assume that no compaction occurred in the dense horizons (bog transition zone: 1.29±0.04 g/cm^3, bog mineral horizon: 1.74±0.01 g/cm^3, fen transition zone: 1.97±0.2 g/cm^3, fen mineral horizon: 1.72±0.01 g/cm^3).

Thus, where no compaction was assumed (Supplementary Figure 9), the stock was calculated without compaction consideration i.e. thickness used for the calculation is as reported by depth intervals in the main text:

$$Stock = bulk\ density * content * layer\ thickness$$

with stock in mg/cm^{-2}, bulk density in g cm^{-3}, content in mg g^{-1} and actual layer thickness in the core in cm.

For the horizons where compaction was assumed (Supplementary Figure 9), a compaction factor was calculated from the difference between the actual core depth (as reported in the text) and the core hole (compaction factor for palsa organic horizon and palsa transition zone 2.31, for bog organic horizon 5.00 and for fen organic horizon 8.25). The compaction-corrected stock was calculated as follows:

$$Stock = compaction\ factor * bulk\ density * content * layer\ thickness$$

with stock in mg/cm^{-2}, bulk density in g cm^{-3}, content in mg g^{-1} and actual layer thickness in the core in cm.

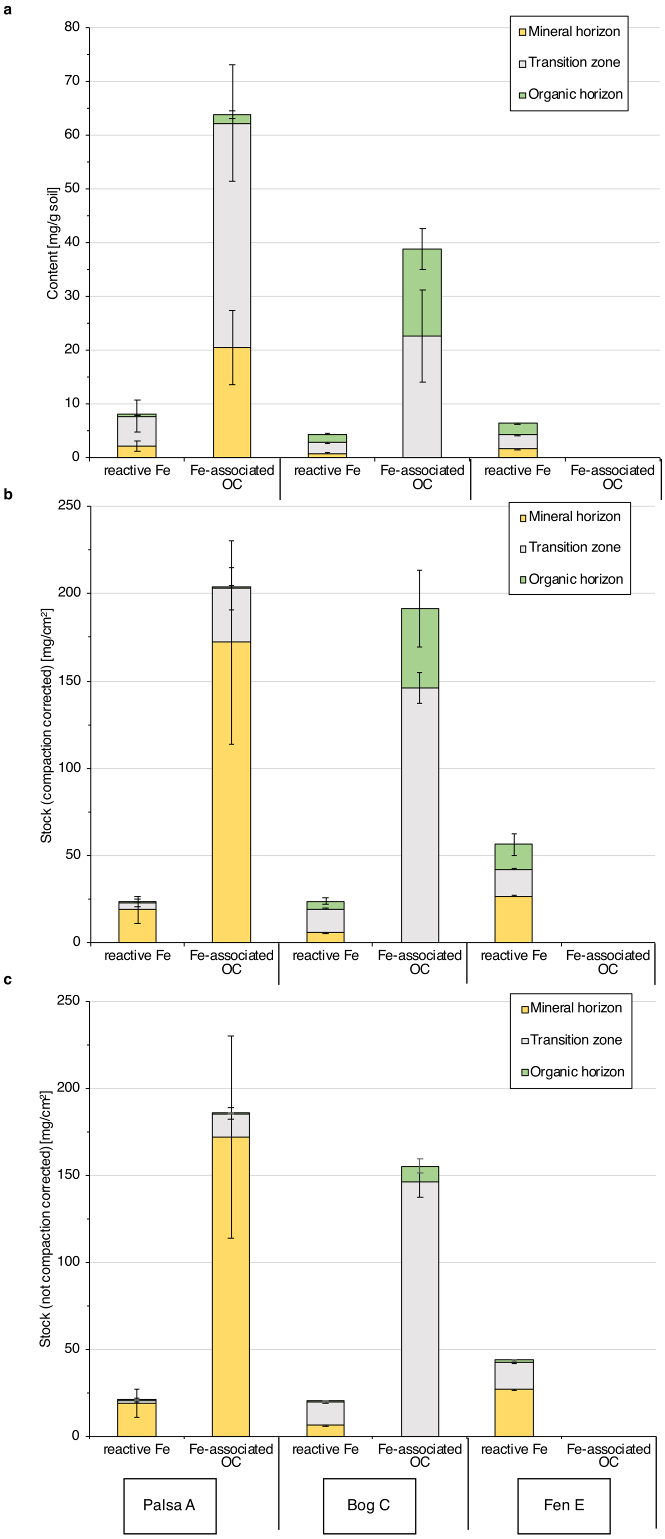

Supplementary Figure 9. Calculated stocks (mg/cm^2) versus absolute amounts (mg/g) of reactive iron (Fe) and reactive Fe-associated organic carbon (OC). a, Averaged absolute amounts of reactive Fe and associated organic carbon in mg per g soil per horizon (green: organic horizon, grey: transition zone, yellow: mineral horizon) for palsa, bog and fen. Error bars represent a combined standard deviation of the absolute values per depth (see Table

S1) of duplicate extractions per horizons of Palsa A, Bog C and Fen E. b, Calculated average stock of reactive Fe and reactive Fe-associated OC in mg per cm^2, compaction corrected. Green represents the organic horizon, grey the transition zone and yellow the mineral horizon in Palsa E, Bog C and Fen E. Error bars represent a combined standard deviation of the absolute values, bulk density and compaction per horizons of Palsa A, Bog C and Fen E. c, Calculated average stock of reactive Fe and reactive Fe-associated OC in mg per cm^2 without assuming compaction. The reported depth intervals in the main text were used to calculate the stock. Green represents the organic horizon, grey the transition zone and yellow the mineral horizon in Palsa E, Bog C and Fen E. Error bars represent a combined standard deviation of the absolute values and the bulk density per horizons of Palsa A, Bog C and Fen E.

Supplementary Figure

Additional elements (P, S and Al) appearing with Fe minerals along the thaw gradient

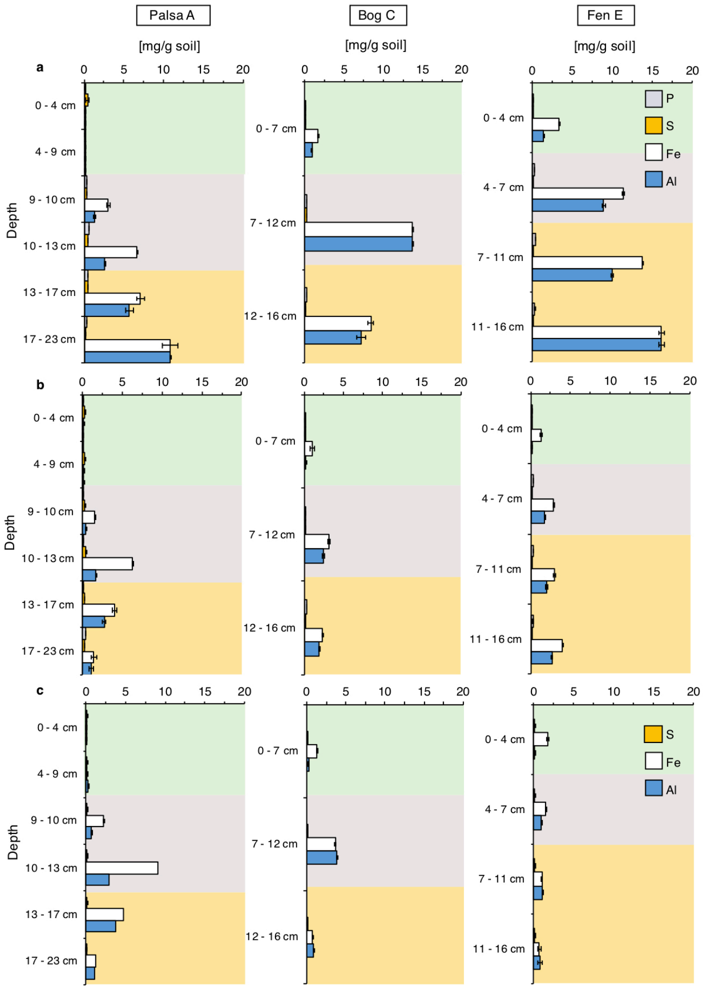

Supplementary Figure 10. Other elements (phosphorous (P), sulphur (S), iron (Fe) and aluminum (Al)) in extracts of Palsa A, Bog C and Fen E. a, 6M hydrochloric acid (HCl), b, hydroxylamine-HCl and c, sodium pyrophosphate extracted. Fe, P and S concentrations were measured with ICP-MS (inductively coupled plasma mass spectrometry). Fe and Al concentrations were analyzed using MP-AES (microwave plasma atomic emission spectroscopy). The illustrated Fe values here are measured by MP-AES. The slightly different Fe concentrations by the different analytical approaches (ferrozine assay, MP-AES and ICP-MS) are shown in Supplementary Figure 4. The green box marks the organic horizon, grey box the transition zone and yellow box the mineral horizon. Errors indicate the range of duplicate analyses of each layer in each thaw stage.

Supplementary Figure

Replication of nanoSIMS analysis (4 representatives per soil layer)

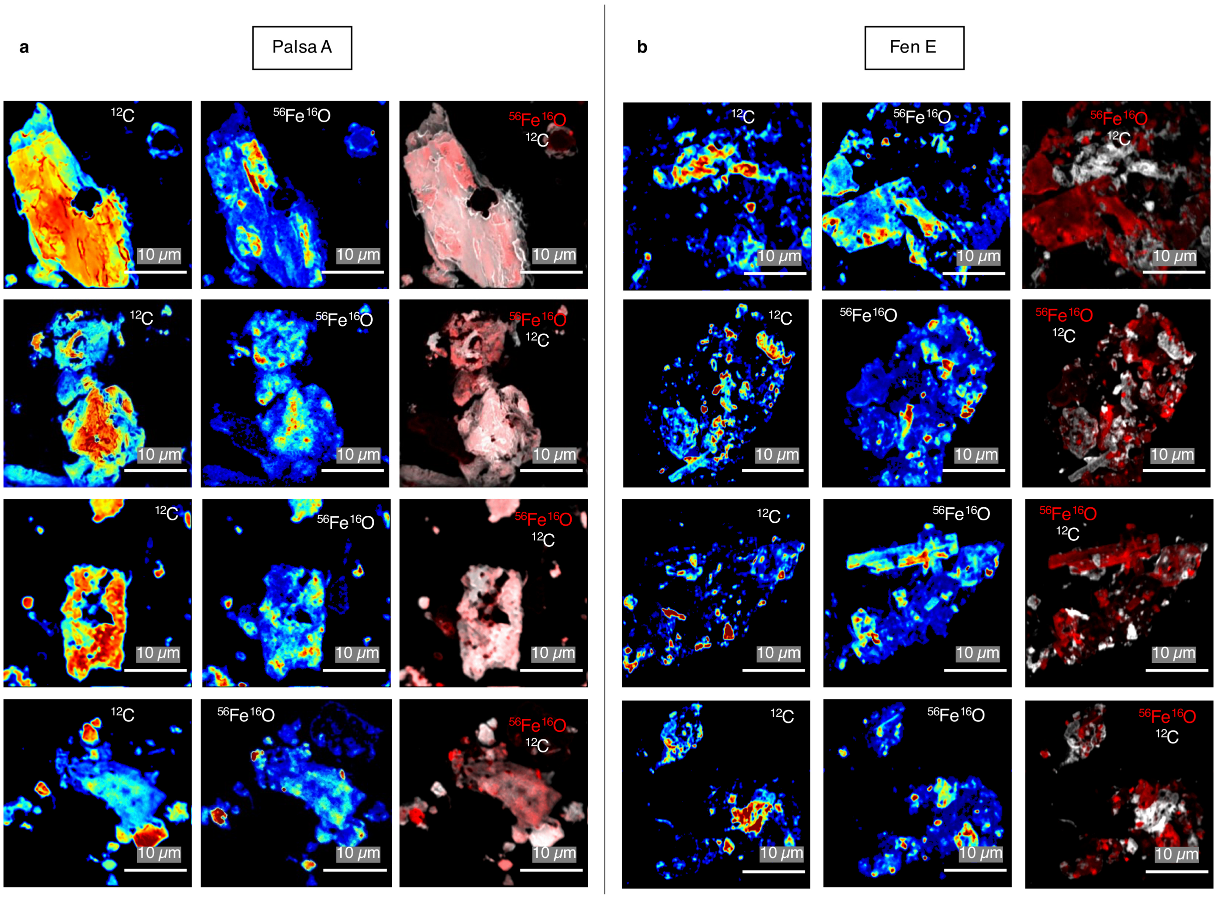

Supplementary Figure 11. Four representative particles of the fine fraction of a, Palsa A analyzed with nanoSIMS showing a close spatial distribution of Fe and C. Seven detectors were used during nanoSIMS measurements for ^{12}C, $^{56}Fe^{16}O$, ^{16}O, $^{12}C^{14}N$, ^{31}P, ^{32}S and $^{27}Al^{16}O$. ^{12}C and $^{56}Fe^{16}O$ are shown for four representative fine particles plus ^{12}C and $^{56}Fe^{16}O$ distributions, overlain in a single image on the left. Four representative particles of the fine fraction of b, Fen E analyzed with nanoSIMS showing organic-free iron minerals. Seven detectors were used during nanoSIMS measurements for ^{12}C, $^{56}Fe^{16}O$, ^{16}O, $^{12}C^{14}N$, ^{31}P, ^{32}S and $^{27}Al^{16}O$. ^{12}C and $^{56}Fe^{16}O$ are shown for four representative fine particles plus ^{12}C and $^{56}Fe^{16}O$ distributions, overlain in a single image on the left.

Supplementary Method 2

Different Fe analysis of the extracts to rule out matrix effects

Different analytical approaches (ferrozine assay, MP-AES, ICP-MS) have been used to determine Fe in the extracts, to rule out matrix effects and to determine additional elements in the extracts (Supplementary Figure 12). ICP-MS was also used to measure sulphur (S) and phosphorous (P) (Supplementary Figure 8). MP-AES was also used to determine aluminum concentrations in the extracts (Al) (Supplementary Figure 8). For the ferrozine assay, the calibration curves were $r^2 > 0.999$, and the standard deviations of the triplicate analyses were <1%. For the ICP-MS, the calibration curves were $r^2 > 0.999$, and the standard deviations of the triplicate analyses were <5%. For the MP-AES, the calibration curves were $r^2 > 0.993$ and the standard deviations of the triplicate analysis were <10%. We are aware of differences between the iron values. However, because the values only vary slightly, the ferrozine values had a higher accuracy (<1%) and sodium dithionite citrate was not measured with ICP-MS and MP-AES due to citric formation after acidification, we decided to use the data from the ferrozine assay.

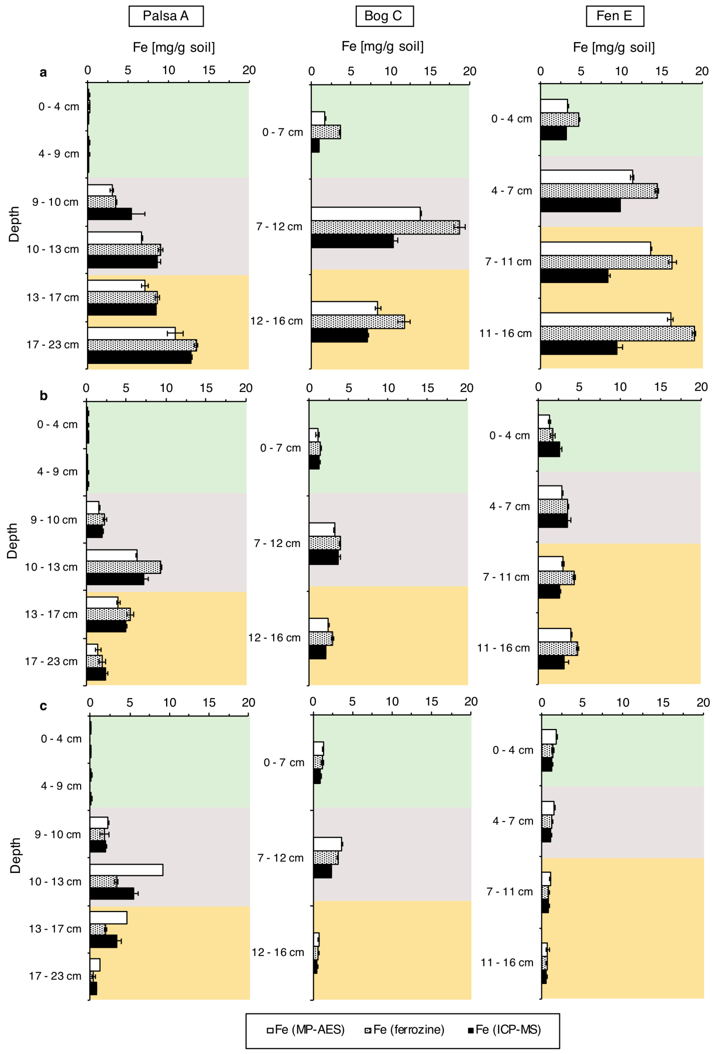

Supplementary Figure 12. Fe analysis by MP-AES (microwave plasma atomic emission spectroscopy), ferrozine and ICP-MS (inductively coupled plasma mass spectrometry) analysis of Palsa A, Bog C and Fen E: a, 6M hydrochloric acid (HCl) extraction, b, hydroxylamine-HCl extraction and c, sodium pyrophosphate extraction. Due to citric acid formation after acidification, the sodium dithionite citrate extract was not measured with the more sensitive MP-AES and ICP-MS instrument. For the 6M HCl and hydroxylamine-HCl extracts, the MP-AES iron values are slightly lower than the ferrozine iron values due to matrix interference during MP-AES measurements of the extracts (HCl). For the sodium pyrophosphate extract, the MP-AES iron values are slightly higher than the ferrozine iron values. This could be explained by the dark color of the extracts which disturb the spectrophotometric measurement during ferrozine complexation. After blank subtraction this can result in lower iron concentrations determined by the ferrozine assay. The ICP-MS iron values are close to the ferrozine values

(122±54% similarity), except for one extraction (6M HCl extraction) and two horizons (transition zone and mineral horizon of bog and fen). Errors indicate the range of duplicate analyses of each layer in each thaw stage.

Supplementary References

1 Wagai, R. & Mayer, L. M. Sorptive stabilization of organic matter in soils by hydrous iron oxides. *Geochim Cosmochim Ac* **71**, 25-35 (2007).

2 Kaiser, K. & Guggenberger, G. Sorptive stabilization of organic matter by microporous goethite: sorption into small pores vs. surface complexation. *Eur J Soil Sci* **58**, 45-59 (2007).

3 Coward, E. K., Thompson, A. T. & Plante, A. F. Iron-mediated mineralogical control of organic matter accumulation in tropical soils. *Geoderma* **306**, 206-216 (2017).

4 Holmgren, G. G. A Rapid Citrate-Dithionite Extractable Iron Procedure. *Soil Sci Soc Am Pro* **31**, 210-211 (1967).

5 Loeppert, R. H., Inskeep, W. P. Iron. *In: Sparks DL (ed) Methods of soil analysis. Part 3, chemical methods. Soil Sci. Soc. Am. Book Series 5, Madison,* WI (1996).

6 Wagai, R., Mayer, L. M., Kitayama, K. & Shirato, Y. Association of organic matter with iron and aluminum across a range of soils determined via selective dissolution techniques coupled with dissolved nitrogen analysis. *Biogeochemistry* **112**, 95-109 (2013).

7 Lalonde, K., Mucci, A., Ouellet, A. & Gelinas, Y. Preservation of organic matter in sediments promoted by iron. *Nature* **483**, 198-200 (2012).

8 Mehra, O. P. & Jackson, M. L. Iron oxide removal from soils and clays by a dithionite-citrate system buffered with sodium bicarbonate. *Clays Clay Min.* **7**, 317-327 (1958).

9 Ryan, J. N. & Gschwend, P. M. Extraction of iron oxides from sediments using reductive dissolution by titanium(III). *Clays and Clay Min* **39**, 509-518 (1991).
